# Multiple Right Ventricular Haemangiomas

**DOI:** 10.7759/cureus.36570

**Published:** 2023-03-23

**Authors:** Emeka B Kesieme, Keith G Buchan

**Affiliations:** 1 Cardiothoracic Surgery, Aberdeen Royal Infirmary, Aberdeen, GBR

**Keywords:** hemangioma, haemangioma, right ventricular haemangioma, right ventricle, haemangiomata, cavernous haemangioma, ventricular haemangioma, cardiac tumours

## Abstract

Right ventricular haemangiomas are rare benign tumours, usually solitary and commonly located in the right heart.

We report a 49-year-old female who presented with four masses in the right ventricle, three arising from the right ventricular free wall and one arising from the anterior leaflet of the tricuspid valve. She subsequently underwent total excision of the tumours and an anteroinferior commissuroplasty for severe tricuspid regurgitation complicating the excision. Histology confirmed cavernous haemangioma.

Solitary haemangioma of the right ventricle has been reported severally in the literature but, to the best of our knowledge, this is the first report of multiple right ventricular haemangiomas.

## Introduction

Primary cardiac tumours are uncommon and are seen in 0.01-0.03% of autopsies [1]. They usually present with one or more of the following: features from intracardiac obstruction, systemic embolization, and systemic or constitutional symptoms [1].

Haemangiomas are rare benign tumours, accounting for about 2.8% of all primary resected tumours of the heart [2]. They may be asymptomatic and discovered incidentally or they may present with symptoms related to their size and anatomic location within the cardiac chamber. Symptoms may also be non-specific and may include palpitations, decreased exercise tolerance, and shortness of breath [3].

Any cardiac chamber or layer of the heart wall may be involved. The right atrium has been reported to be the most common location in 26.2% of cases [3]. In an analysis done by Li et al., the right heart (right atrium, right ventricle, tricuspid valve, and pulmonary valve) was involved in 44.1% of cardiac haemangiomas while the left heart (left atrium, left ventricle, mitral valve, and aortic valve) was involved in 39.5% of cases [3].

Histologic variants include capillary (capillary-like vessel), cavernous (multiple thin-walled vessels), and arteriovenous type (dysplastic malformed arteries and veins) [4]. Definitive treatment is complete surgical excision of the tumour.

We report the first case of multiple right ventricular haemangiomas treated with surgical excision after a diagnosis of intracardiac masses was made by echocardiography and cardiac magnetic resonance imaging.

## Case presentation

A 49-year-old female presented to the cardiology department with dizziness, palpitation, and an episode of collapse. A cardiovascular examination revealed a pulse of 84 beats/minute, which was regular. Blood pressure was normal (104/72 mmHg). Jugular venous pressure was not elevated and no heart murmurs were heard. Chest examination was normal. ECG and chest X-ray were normal.

**Figure 1:**
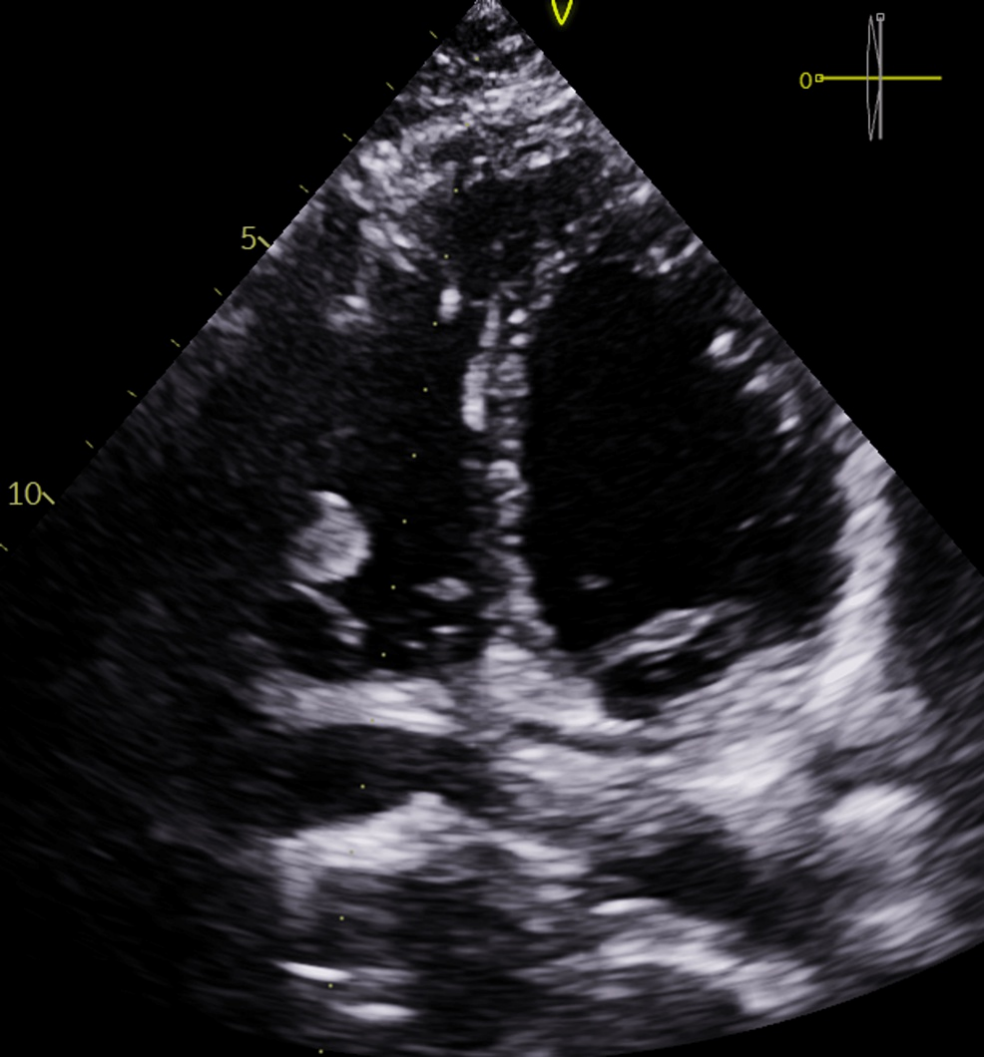
Image showing ventricular mass

**Figure 2:**
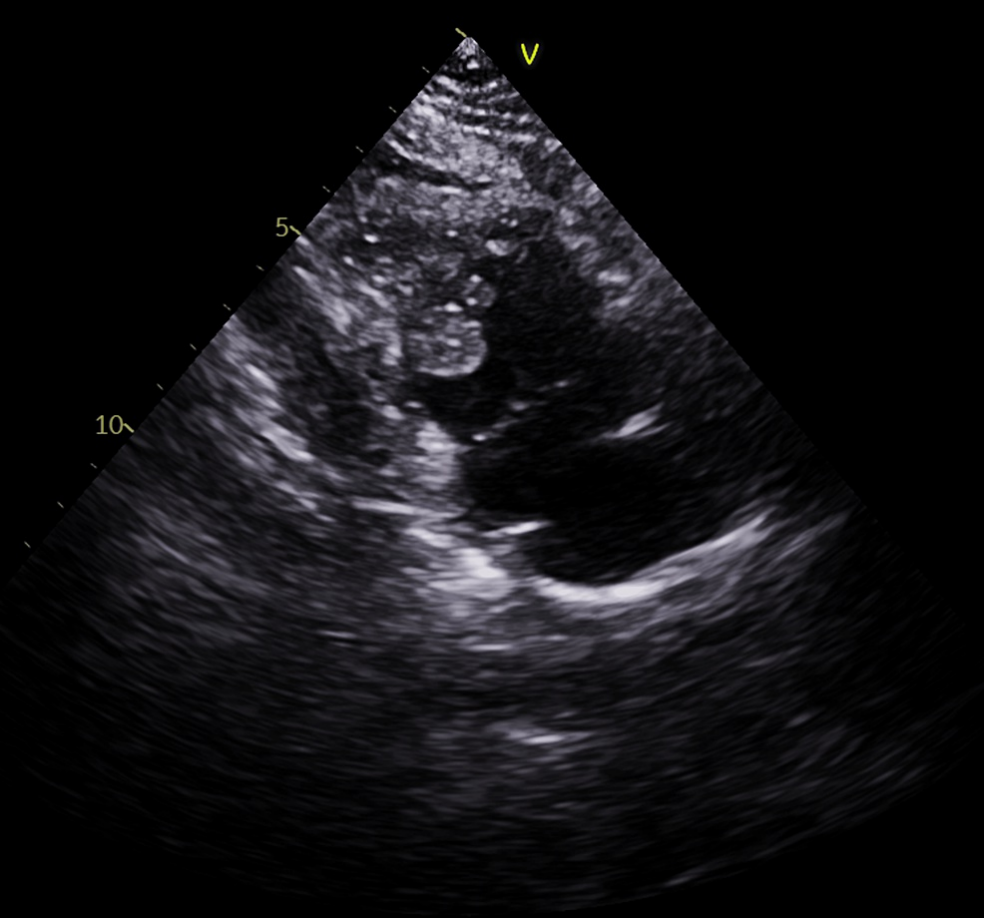
Masses attached to the right ventricular free wall

Echocardiography revealed two separate masses measuring 1.15 x 1.36 cm^2^ and 0.77 x 0.58 cm^2^, respectively, seen attached to the right ventricular free wall. No obstruction or turbulence was noted. The valves were essentially normal, and the left and right ventricles had a good systolic function (Figures 1, 2).

**Figure 3:**
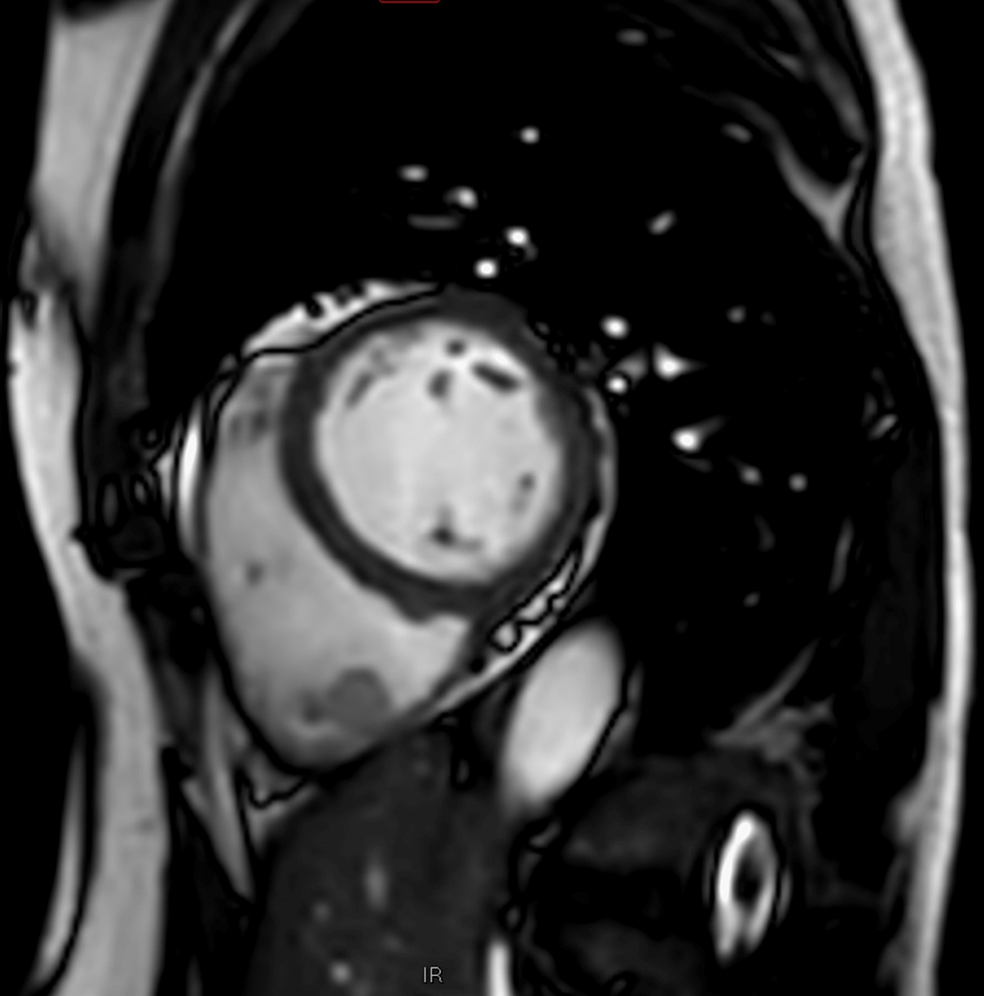
T1-weighted scan of the right ventricular mass

**Figure 4:**
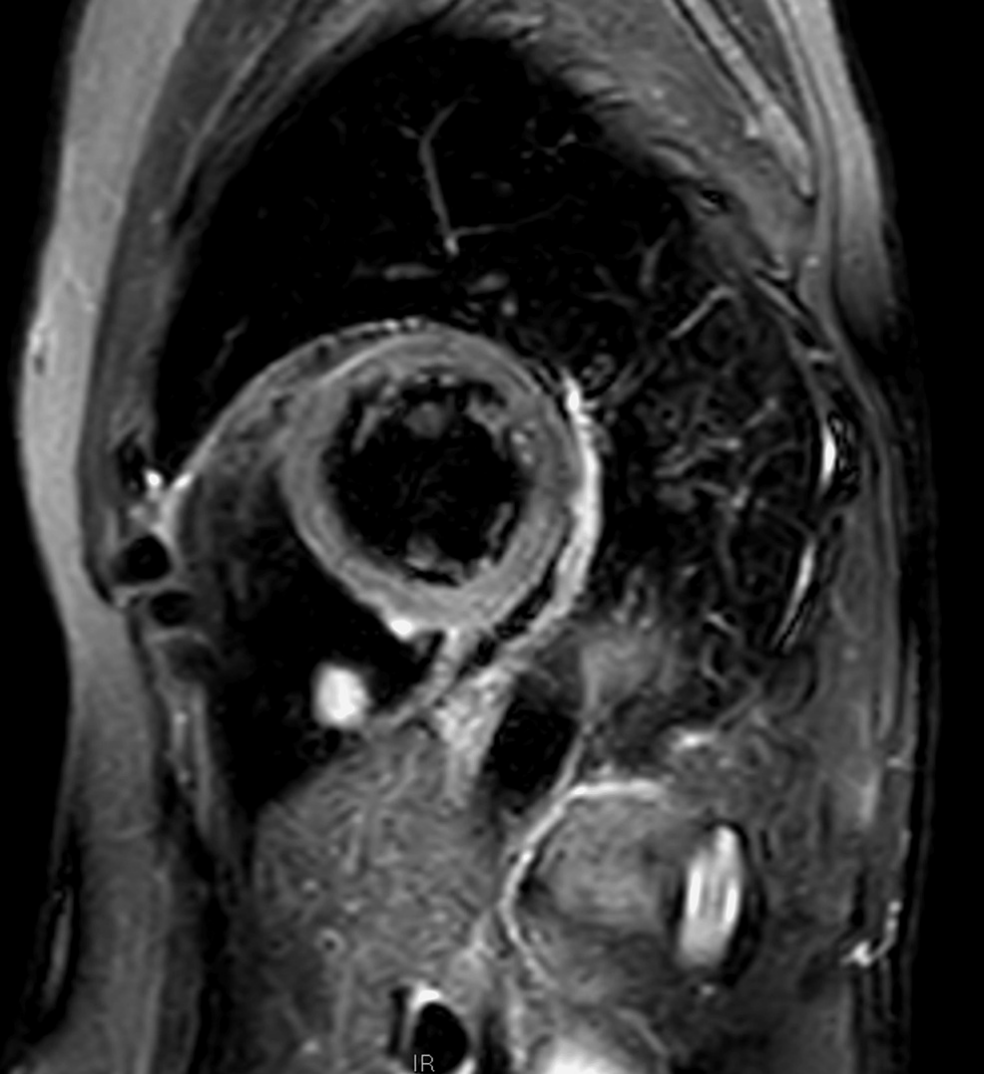
T2-weighted scan of the right ventricular mass

Cardiac magnetic resonance imaging revealed a round well-circumscribed mass in the vicinity of the posterior wall of the right ventricle. The mass was mobile, pedunculated, and did not infiltrate or penetrate the myocardial wall. It had the same structure as the right ventricular myocardium on the T1-weighted scan but was bright on the T2-weighted scan (Figures 3, 4).

Coronary angiography revealed no lesion in the coronary arteries and no tumour blush.

**Figure 5:**
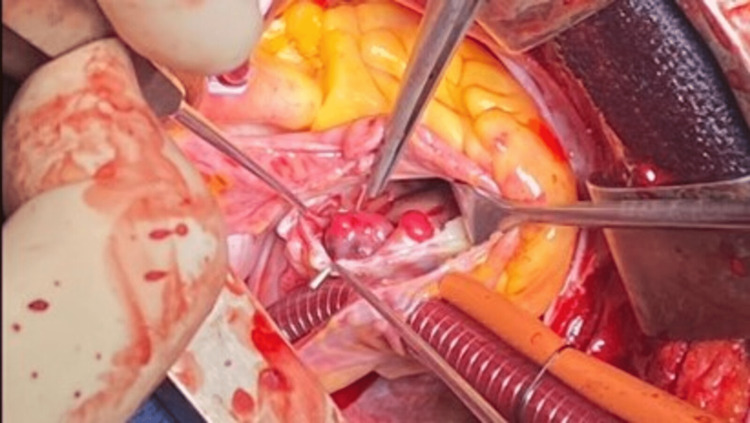
Right ventricular haemangiomas

She was prepared for tumour resection. A median sternotomy was performed. Cardiopulmonary bypass was established through ascending aortic cannulation and bicaval drainage with core temperature cooling to 34^_°_^C. A right atriotomy was performed and the right ventricle was accessed through the tricuspid valve. Four tumours were seen (Figure 5).

The largest tumour was attached to the right ventricular free wall. The second largest was arising from the anterior leaflet of the tricuspid valve. Two other smaller lesions were also arising from the right ventricular free wall. Removal of the lesion attached to the tricuspid valve involved severing the principal chord. The other lesions were separated from the right ventricular free wall using sharp dissection. Water testing of the tricuspid valve revealed severe regurgitation. This was due to the loss of chordal support for the anteroinferior commissure. A competent tricuspid valve was achieved after an anteroinferior commissuroplasty using a 5/0 Vascufil monofilament suture.

Tumour histology revealed well-circumscribed, non-encapsulated lesions comprising irregular, small, medium, as well as large vascular spaces lined by a single layer of plump endothelial cells. Mixed epithelioid spindle cells were seen with surrounding connective tissue associated with chronic inflammation and haemosiderin-laden macrophages. There was no evidence of atypia or malignancy. On immunohistochemistry, the vascular lining stained positively with CD31, CD34, FLI1, and ERG. D2-40, S100, and human herpesvirus 8 (HHV8) were negative. Ki-67 showed low proliferative activity. Features were consistent with cavernous haemangioma.

Serial echocardiography follow-up over a period of one year showed no evidence of recurrence.

## Discussion

Multiple intracardiac masses have been rarely reported [5,6]. Differential diagnoses may include thrombus, vegetations, myxomas, lymphoma, and malignant primary and secondary metastatic tumours [5,6]. Multimodality imaging such as echocardiography, cardiac computed tomography scan, and cardiac magnetic resonance imaging can be very useful in making a presumptive diagnosis before intervention in patients with multiple intracardiac masses. Thrombi are characteristically discrete, homogenous in appearance, do not take up the contrast, and the patient may present with risk factors for thrombus formation like atrial fibrillation and biventricular heart failure [6]. They are also more likely to be attached to an area of abnormal wall motion, which may either be akinetic or dyskinetic [7]. Cardiac lymphoma typically presents as ill-defined infiltrative masses or as nodular masses mainly on the right side of the heart, particularly the right atrium [7]. Multiple intracardiac myxomas are a component of the Carney complex. They tend to be familial, occur at an earlier age, and have a high risk of recurrence. Myxomas are often pedunculated [1,7]. Enhancement can help distinguish thrombi from myxoma and malignant and highly vascular tumours. Thrombi do not enhance with contrast. Myxomas tend to be partially enhanced, whereas malignant and hypervascular tumours demonstrate hyperenhancement with contrast [7,8]. Valvular masses should raise suspicion for vegetations [7].

There are several case reports of haemangioma in the right ventricle, but all of them were solitary lesions [9-11]. Haemangiomas have also been reported in different locations in the right ventricle and there may be involvement of the subvalvular apparatus [4,9,10]. In this patient, the tumours were attached to the free wall of the right ventricle and one of them was attached to the principal cord of the anteroinferior commissure of the tricuspid valve. Domoto et al. reported a case involving the anterior right ventricular wall and tricuspid valve with the deep involvement of the anterior papillary muscle [4].

The size of these tumours may vary from small to large. Symptoms may depend on the size and location of the tumour. Chest pain may be due to coronary steal, exertional dyspnoea, and murmur of pulmonary stenosis in tumours located in the right ventricular outflow tract. Conduction disturbances can result from tumours involving the atrioventricular node [10,11].

Transthoracic echocardiography (TTE) and cardiac magnetic resonance imaging are important diagnostic modalities. The tumour usually presents as a hyperechoic lesion on TTE, with intermediate density on T1-weighted imaging and hyperdensity on T2-weighted imaging. Coronary angiography may show tumour-feeding arteries and tumour blushes; however, none was observed in this patient [11].

In addition to tumour resection, there may be a need for surgical intervention for associated injury to the tricuspid valve or its subvalvular apparatus. An anteroinferior commissuroplasty of the tricuspid valve was performed in this case. Domoto et al. performed applied cryoablation to the resected marginal side of the right ventricle and tricuspid valve repair for their patient [4].

It is important to monitor recurrence, as it has been reported in some cases [12].

## Conclusions

In conclusion, we have reported the first case of multiple haemangiomas of the right ventricle that underwent successful excision. This case suggests the action of an as-yet unidentified genetic field change affecting the right ventricular substrate, which was responsible for the simultaneous development of multiple haemangiomas.
